# Prognostic value of β-Arrestins in combination with glucocorticoid receptor in epithelial ovarian cancer

**DOI:** 10.3389/fonc.2023.1104521

**Published:** 2023-03-10

**Authors:** Ji-Won Ryu, Ha-Yeon Shin, Hyo-Sun Kim, Gwan Hee Han, Jeong Won Kim, Hae-Nam Lee, Hanbyoul Cho, Joon-Yong Chung, Jae-Hoon Kim

**Affiliations:** ^1^ Department of Obstetrics and Gynecology, Yonsei University College of Medicine, Seoul, Republic of Korea; ^2^ Department of Obstetrics and Gynecology, Kyung Hee University Hospital at Gangdong, Seoul, Republic of Korea; ^3^ Department of Pathology, Kangnam Sacred Heart Hospital, Hallym University College of Medicine, Seoul, Republic of Korea; ^4^ Department of Obstetrics and Gynecology, Catholic University of Korea Bucheon St. Mary’s Hospital, Bucheon, Republic of Korea; ^5^ Department of Obstetrics and Gynecology, Gangnam Severance Hospital, Yonsei University College of Medicine, Seoul, Republic of Korea; ^6^ Institute of Women’s Life Medical Science, Yonsei University College of Medicine, Seoul, Republic of Korea; ^7^ Molecular Imaging Branch, National Cancer Institute, Center for Cancer Research, National Institutes of Health, Bethesda, MD, United States

**Keywords:** β-arrestin, glucocorticoid receptor, epithelial ovarian cancer, prognostic power, steroid hormones

## Abstract

Hormones may be key factors driving cancer development, and epidemiological findings suggest that steroid hormones play a crucial role in ovarian tumorigenesis. We demonstrated that high glucocorticoid receptor (GR) expression is associated with a poor prognosis of epithelial ovarian cancer. Recent studies have shown that the GR affects β-arrestin expression, and vice versa. Hence, we assessed the clinical significance of β-arrestin expression in ovarian cancer and determined whether β-arrestin and the GR synergistically have clinical significance and value as prognostic factors. We evaluated the expression of β-arrestins 1 and 2 and the GR in 169 patients with primary epithelial ovarian cancer using immunohistochemistry. The staining intensity was graded on a scale of 0–4 and multiplied by the percentage of positive cells. We divided the samples into two categories based on the expression levels. β-arrestin 1 and GR expression showed a moderate correlation, whereas β-arrestin 2 and GR expression did not demonstrate any correlation. Patients with high β-arrestin 1 and 2 expression exhibited improved survival rates, whereas patients with low GR expression showed a better survival rate. Patients with high β-arrestin 1 and low GR levels had the best prognosis among all groups. β-arrestin is highly expressed in ovarian cancer, suggesting its potential as a diagnostic and therapeutic biomarker. The combination of β-arrestin and GR demonstrated greater predictive prognostic power than GR expression alone, implicating another possible role in prognostication.

## 1 Introduction

The 5-year relative survival rate of patients with various cancers has increased by 20% over the last 30 years (1). Despite this improvement, the survival rate of patients with ovarian cancer remains limited compared to that of patients with other cancers (2, 3). Ovarian cancer is the fifth leading cause of cancer-related deaths among women worldwide and the most lethal of all gynecological cancers (4). Although patients with early-stage disease have a survival rate of approximately 90%, most women are diagnosed in stage III/IV, and more than 75% of them die from this disease (5). Cytoreductive surgery and chemotherapy, the standard treatments for ovarian cancer, are accompanied by a high risk of relapse and resistance to chemotherapy (6). Although trials to overcome these limitations, including the use of bevacizumab, poly(ADP-ribose) polymerase inhibitors, and programmed cell death protein 1 blockers, are ongoing, early results indicate that these therapies cannot effectively reduce disease-specific mortality, despite their success against other advanced cancers (7–10). Therefore, the mechanisms that can overcome chemotherapy resistance and relapse in ovarian cancer must be better defined.

Ovarian cancer is a hormone-dependent malignancy, and its progression is affected by steroid hormones and their receptors (8). Nevertheless, the efficacy of hormone therapy in ovarian cancer remains limited due to the various histopathological types, the diversity of hormone receptor expression, and the lack of molecular markers (8). However, the dysregulation of steroid hormone receptor (SHR) signaling in cancer can be exploited as a treatment strategy (11). Hence, we investigated the expression of SHRs and their clinical significance in patients with ovarian cancer and reported that the expression of the GR modifies the role of the progesterone receptor (PR) and affects the androgen receptor (AR) (12). The GR is a major transcription factor regulating gene expression after glucocorticoid binding. Glucocorticoid-mediated transcription regulates selective gene transcription, although the mechanism remains nebulous (13). GR activation by dexamethasone inhibits the cell death in breast, cervical, and ovarian cancer cell lines and xenograft models (14). Currently, the GR is not used as a therapeutic or diagnostic marker in clinical practice, although high GR expression is associated with a poor prognosis of ovarian cancer (15). Co-factors or co-regulators of the GR have to be identified to unravel its role in ovarian cancer.

Recent studies have demonstrated the mutual relationship between β-arrestins and the GR (13, 16–18). The GR enhances the expression of β-arrestins, and in turn, β-arrestins are highly correlated with GR stability, affecting GR protein turnover (16). β-arrestins are scaffolding proteins involved in the negative signaling of G-protein-coupled receptors, affecting cell proliferation, cytoskeletal rearrangement, and cell motility (19). β-arrestins are also involved in cancer cell phenotypes, such as cancer cell migration, invasion, and metastasis, in various cancers (20–22). Additionally, β-arrestins play ambivalent roles in promoting or inhibiting tumor growth (23), with their overall effect depending on the tumor microenvironment and interactions with various receptors or signaling systems (24, 25). Studies on the expression of β-arrestins and their effects on prognosis are being conducted in several tumor types, but the role of β-arrestins in ovarian cancer treatment response and survival is not yet well understood. In the present study, we aimed to investigate the expression of β-arrestins in ovarian cancer and assess their prognostic value in combination with GR expression.

## 2 Materials and methods

### 2.1 Patients and tumor specimens

We obtained 169 formalin-fixed paraffin-embedded surgical specimens of ovarian cancer and 66 normal ovarian epithelial tissues from the Departments of Obstetrics and Gynecology of Gangnam Severance Hospital and Yonsei University College of Medicine, and the Korea Gynecologic Cancer Bank through the Bio and Medical Technology Development Program of the Ministry of Education, Science and Technology, Korea (NRF-2017M3A9B8069610). The surgical specimens were tissues from patients with ovarian cancer who underwent surgery at the Gangnam Severance Hospital and Bucheon St. Mary’s Hospital between 1997 and 2012. The exclusion criteria included a diagnosis of recurrent ovarian cancer, peritoneal and fallopian tube cancers, or any other invasive cancers as well as the use of immunosuppression therapies. A retrospective chart review was conducted to obtain patients’ clinical information, including age and disease stage at the time of diagnosis (according to FIGO stage), cell type, tumor grade, tissue differentiation status, surgery date, blood test results before surgery, and sensitivity to chemotherapy. Recurrence and treatment response were determined using Response Evaluation Criteria in Solid Tumors (RECIST; version 1.1) on computed tomography scans. Patient status was assessed at the last follow-up visit, and patients lost to follow-up were interviewed *via* telephone. Recurrence-free survival (RFS) was calculated from the date of surgery to the date when recurrence was confirmed or the last follow-up visit in cases without recurrence. Overall survival (OS) was calculated from the date of surgery to the date of confirmed death or the last follow-up visit for patients who were alive. We used all patients’ tissues and medical records after disseminating notice to the patients and obtaining their informed consent according to the guidelines of the Gangnam Severance Hospital institutional review board (IRB 3-2021-0361, Seoul, Korea).

### 2.2 Tissue microarray-based immunohistochemistry

We prepared human ovarian tissue microarrays as previously reported (12). In brief, we sectioned the formalin-fixed paraffin-embedded tumor tissue blocks to a thickness of 5 μm. The sections were deparaffinized in xylene and rehydrated using a graded ethanol series. Antigen retrieval was performed by incubating the samples in antigen retrieval buffer, pH 6.0 (Dako, Carpinteria, CA, USA) for β-arrestin 1 and pH 9.0 (Dako) for β-arrestin 2 for 20 min. We quenched endogenous peroxidase activity with 3% hydrogen peroxide for 15 min. Protein blocking was performed for 20 min.

The sections were incubated with rabbit monoclonal anti-β-arrestin 1 (Cat. No. ab32099; 1:2,000; Abcam, Cambridge, MA, USA) and goat polyclonal anti-β-arrestin 2 (Cat. No. ab31294; 1:400; Abcam) at room temperature for 1 h. Antigen–antibody reactions were detected with EnVision+ Rabbit-HRP (Dako) or LSAB (Dako) and visualized with 3,3-diaminobenzidine (Dako). The sections were counterstained with hematoxylin and then mounted. Appropriate positive and negative controls were run concurrently.

### 2.3 Evaluation of immunohistochemical staining

The stained tissue microarray sections were scanned with a high-resolution optical scanner (NanoZoomer 2.0 HT; Hamamatsu Photonic K.K., Hamamatsu City, Japan) at 20× objective magnification (0.5-mm resolution). The scanned sections were analyzed using VIS Image Analysis Software, version 4.5.1.324 (Visiopharm, Hørsholm, Denmark). β-arrestin 1 was stained in 149 ovarian cancer specimens and 49 non-adjacent normal epithelial tissues, whereas β-arrestin 2 was stained in 114 ovarian cancer specimens and 34 non-adjacent normal epithelial tissues. The expression of the GR was examined based on a study at our institute published in 2021 (12). Staining intensity was scored on a scale of 0–4 (0 = negative, 1 = weak, 2 = moderate, 3 = strong), and immunoreactivity score was calculated by multiplying the intensity score with the percentage of positive cells (possible range, 0–300). The Contal & O’Quigley method was used to obtain the optimal cutoffs to divide the expression levels into two groups (OS cut-off histoscore: β-arrestin 1 = 28.385, β-arrestin 2 = 69.632, RFS cut-off histoscore: β-arrestin 1 = 31.96, β-arrestin 2 = 69.632). For the GR, the cut-off values from the 2021 study were used OS and recurrence free survival (RFS) cut-offs = 6.85] (12).

### 2.4 *In silico* analysis of β-arrestin and GR expression

Data from the Gene Expression Omnibus (GEO), the Gene Expression Profiling Interactive Analysis (GEPIA), and The Cancer Genome Atlas (TCGA) were used in this study. The datasets GSE14407 (serous type; normal = 12, cancer = 12), GSE26712 (serous type; normal = 10, cancer = 185), and GSE29450 (clear cell type; normal = 12, cancer = 20) were used. Correlations among β-arrestin 1, β-arrestin 2, and GR expression were determined based on TCGA and the genotype-tissue expression data in the GEPIA. Kaplan–Meier survival curves were generated using TCGA data in cBioPortal (http://www.cbioportal.org).

### 2.5 Statistical analysis

β-arrestin 1, β-arrestin 2, and GR expression data were statistically analyzed using Mann–Whitney or independent *t*-test, as appropriate. The chi-square test or Fisher’s exact test was used for patient characteristics. Survival was analyzed using the log-rank test with the cut-off values determined using the Contal & O’Quigley method and Kaplan–Meier plots. Spearman correlation was used to assess the relationships among β-arrestins 1, β-arrestin 2, and the GR. Cox proportional hazard models were used to estimate the Hazard Ratios(HRs) and confidence intervals (CIs) for univariate and multivariate models. We performed a C-index comparison to determine whether the predictive power of the models would increase when independent variables were combined. We performed statistical analyses using SPSS version 26.0 (IBM SPSS Statistics, Chicago, IL, USA), SAS version 9.4 (SAS Institute, Cary, NC, USA), and R version 4.0.3 (http://www.r-project.org). Statistical significance was set at *P* < 0.05.

## 3 Results

### 3.1 Clinicopathological characteristics

The mean age of the 169 patients was 51.7 ( ± 11.9) years. The serous type accounted for the largest proportion of cases (n = 110, 65.1%), followed by the endometrioid type (n = 23, 13.6%), mucinous type (n = 19, 11.2%), clear cell type (n = 13, 7.7%), and transitional cell type (n = 4, 2.4%). Fifty-seven patients (33.7%) had stage I or II tumors at the time of diagnosis, whereas 112 patients (66.3%) were diagnosed with stage III or higher disease. Among the 169 patients, tumor recurrence was recorded in 93 patients, and 61 patients died of ovarian cancer. The mean follow-up period was 94 months (range, 1–385 months, **Supplementary Table S1**).

Patients with clinical stage I ovarian cancer at the time of diagnosis showed the highest β-arrestin 1 expression. In contrast, patients with stage II disease exhibited the lowest β-arrestin 1 expression. Interestingly, β-arrestin 2 expression varied by stage (**Supplementary Figures S1A, B**). Low β-arrestin 1 expression was observed slightly more often than high β-arrestin 1 expression in advanced stages (stages III and IV, *P* = 0.010). However, there were no significant differences in β-arrestin 1’s expression level according to other factors, including age, CA125 level, chemotherapy resistance, cell differentiation at the time of diagnosis, and cell type. β-arrestin 2 expression was not significantly different when considering all clinicopathological characteristics within the cohort. The patients’ clinicopathological characteristics and β-arrestin 1 and 2 expression levels are presented in **Table 1A**.

**Table 1:** A. The association between clinicopathologic features and β-arrestin 1,2 expression.

Patient characteristic	β-Arrestin 1 lown = 74 (%)	β-Arrestin 1 highn = 75 (%)	*P*-value	β-Arrestin 2 lown = 30 (%)	β-Arrestin 2 highn = 84 (%)	*P*-value
Age (years)			0.356			0.534
<50	29 (39.19)	35 (46.67)		13 (43.33)	31 (36.90)	
≥50	45 (60.81)	40 (53.33)		17 (56.67)	53 (63.10)	
CA125 (mmol/L)			0.826			>0.999
<35	12 (16.22)	13 (17.57)		3 (10.00)	10 (11.90)	
≥35	62 (83.78)	61 (82.43)		27 (90.00)	74 (88.10)	
Chemosensitivity			0.589			0.233
Sensitive	57 (85.07)	60 (88.24)		20 (74.07)	67 (85.90)	
Resistant	10 (14.93)	8 (11.76)		7 (25.93)	11 (14.10)	
Stage			0.010*			0.757
I, II	17 (22.97)	32 (42.67)		7 (23.33)	22 (26.19)	
III, IV	57 (77.03)	43 (57.33)		23 (76.67)	62 (73.81)	
Cell type			0.816			0.809
Others	27 (36.49)	26 (34.67)		10 (33.33)	26 (30.95)	
Serous	47 (63.51)	49 (65.33)		20 (66.67)	58 (69.05)	
Grade			0.116			0.200
Well–moderate	31 (43.06)	42 (56.00)		12 (40.00)	44 (53.66)	
Poor	41 (56.94)	33 (44.00)		18 (60.00)	38 (46.34)	

*P < 0.05, chi-square test or Fisher’s exact test. Optimal cut-off points were determined using the Contal & O'Quigley method. The cut-off value for β-arrestin 1 expression was 28.38 and that for β-arrestin 2 expression was 69.93.

**Table:** B. Correlation between clinicopathological characteristics of patients and combined β-arrestin 1 and GR expression.

Patient characteristic	β-Arrestin 1 low^a^ & GR high n = 63 (%)	β-Arrestin 1 low & GR low n = 10 (%)	β-Arrestin 1 high^a^ & GR low n = 9 (%)	β-Arrestin 1 high & GR high n = 66 (%)	*P*-value
Age (years) <50	25 (39.68)	4 (40.00)	5 (55.56)	30 (45.45)	0.794
≥50	38 (60.32)	6 (60.00)	4 (44.44)	36 (54.55)	
CA125 level (mmol/L) <35	8 (12.70)	4 (40.00)	1 (11.11)	12 (18.46)	0.188
≥35	55 (87.30)	6 (60.00)	8 (88.89)	53 (81.54)	
Chemosensitivity Sensitive	46 (82.14)	10 (100.00)	6 (100.00)	54 (87.10)	0.508
Resistant	10 (17.86)	0 (0.00)	0 (0.00)	8 (12.90)	
Stage I, II	13 (20.63)	4 (40.00)	6 (66.67)	26 (39.39)	0.012*
III, IV	50 (79.37)	6 (60.00)	3 (33.33)	40 (60.61)	
Cell type Others	22 (34.92)	5 (50.00)	5 (55.56)	21 (31.82)	0.400
Serous	41 (65.08)	5 (50.00)	4 (44.44)	45 (68.18)	
Grade Well–moderate	24 (38.71)	7 (70.00)	5 (55.56)	37 (56.06)	0.120
Poor	38 (61.29)	3 (30.00)	4 (44.44)	29 (43.94)	

^a^ β-Arrestin 1 expression was categorized as high and low expression according to an optimal cut-off point of 28.38 determined using the Contal & O'Quigley method.

**Table:** C. Correlation between clinicopathological characteristics of patients and combined β-arrestin 2 and GR expression.

Patient characteristic	β-Arrestin 2 low^b^ & GR high n = 28 (%)	β-Arrestin 2 low & GR low n = 0	β-Arrestin 2 high^b^ & GR low n = 10 (%)	β-Arrestin 2 high & GR high n = 72 (%)	*P*-value
Age (years) <50	13 (46.43)	NA	3 (30.00)	27 (37.50)	0.589
≥50	15 (53.57)	NA	7 (70.00)	45 (62.50)	
CA125 (mmol/L) <35	3 (10.71)	NA	2 (20.00)	8 (11.11)	0.726
≥35	25 (89.29)	NA	8 (80.00)	64 (88.89)	
Chemosensitivity Sensitive	20 (80.00)	NA	9 (100.00)	56 (83.58)	0.478
Resistant	5 (20.00)	NA	0 (0.00)	11 (16.42)	
Stage I, II	7 (25.00)	NA	5 (50.00)	17 (23.61)	0.203
III, IV	21 (75.00)	NA	5 (50.00)	55 (76.39)	
Cell type Others	8 (28.57)	NA	5 (50.00)	21 (29.17)	0.390
Serous	20 (71.43)	NA	5 (50.00)	51 (70.83)	
Grade well–moderate	11 (39.29)	NA	6 (60.00)	38 (53.52)	0.362
Poor	17 (60.71)	NA	4 (40.00)	33 (46.48)	

^b^ β-arrestin 2 expression was categorized as high or low according to an optimal cut-off point of 69.93 determined using the Contal & O'Quigley method. NA, not applicable.

### 3.2 Expression of β-arrestins 1 and 2

**Figure 1:**
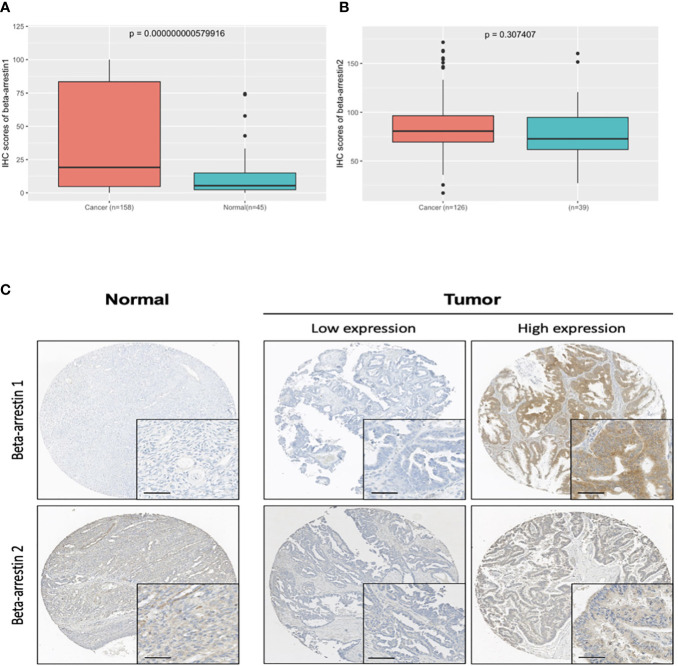
β-Arrestin expression in ovarian cancer tissues. **(A)**, β-Arrestin 1 expression was significantly increased in cancer tissue (*P* = 5.79e−10). **(B)**, β-Arrestin 2 expression in cancer tissue did not differ from that in the nonadjacent normal tissue (*P* = 0.307). **(C)**, Immunohistochemical images of β-arrestins 1 and 2 in ovarian cancer tissues. β-Arrestins 1 and 2 were detected in the cytoplasm. (scale bar: 50 μm).

We performed immunohistochemistry analysis of cancer and the non-adjacent normal tissues to determine the expression pattern of β-arrestin. β-arrestins 1 and 2 were observed in the cytoplasm (**Figure 1C**). For β-arrestin 1, 149 ovarian cancer tissue specimens were interpretable, and 49 non-adjacent normal epithelial tissues were identified using immunohistochemistry. For β-arrestin 2, 114 ovarian cancer tissues were interpretable, and 34 non-adjacent normal epithelial tissues were identified using immunohistochemistry. β-arrestin 1 was significantly more highly expressed in cancer tissues than in the non-adjacent normal tissues (mean histoscore 47.66 vs. 28.85, *P* = 0.02), whereas β-arrestin 2 expression was not significantly different between cancer and non-adjacent normal tissues (mean histoscore 84.48 vs. 79.52, *P* = 0.36) (**Figures 1A, B**). We further analyzed β-arrestin expression using GEO data (**Supplementary Figure S2**). In GSE14407, the expression of β-arrestins 1 and 2 was higher in tumor specimens than in normal tissues, which is consistent with the present study findings (**Supplementary Figure S2A**). However, in GSE26712, β-arrestin 1 was expressed at lower levels in tumor specimens than in normal tissues (**Supplementary Figure S2B**). In GSE16570, containing data of clear cell tumors, β-arrestin 2 was expressed at lower levels in tumor specimens than in normal tissues (**Supplementary Figure S2C**).

### 3.3 Correlations between clinicopathological characteristics and combined β-arrestin and GR expression

We examined the correlations between combined β-arrestin and GR expression and clinicopathological characteristics in the patient cohort (**Tables 1B, C**). The specimens were categorized into high and low expression groups and then further subdivided into four groups. When evaluating β-arrestin 1 and GR expression combined, clinical stage was significant (*P* = 0.012), whereas, age, CA125 level, chemosensitivity, grade, and cell type were not (**Table 1B**). Consistent with prior results, β-arrestin 2 and GR combination did not reveal any significant differences among the clinicopathological characteristics in the cohort (**Table 1C**).

### 3.4 Correlations between β-arrestin and GR expression

β-Arrestin 1 and GR expression showed a moderate correlation (r = 0.274, *P* = 0.001), whereas β-arrestin 2 expression showed no correlation with GR expression (r = 0.044, *P* = 0.660) (**Supplementary Figures S3A, B**). GR expression was higher in stage I than in stages II and III. GR expression and β-arrestin 1 gradually increased from stages II to IV (**Supplementary Figure S1C**). Next, we compared the correlation between β-arrestin and GR expression using public data. Correlations among the expression of GR, β-arrestin 1, and β-arrestin 2 were determined using the GEPIA (**Supplementary Figure S4**). β-arrestin 1 expression had a significant positive correlation with GR expression (r = 0.38, *P* < 0.001). β-Arrestin 2 expression had a moderate correlation with GR expression (r = 0.28, *P* < 0.001).

### 3.5 Prognostic significance of β-arrestins 1 and 2

**Figure 2:**
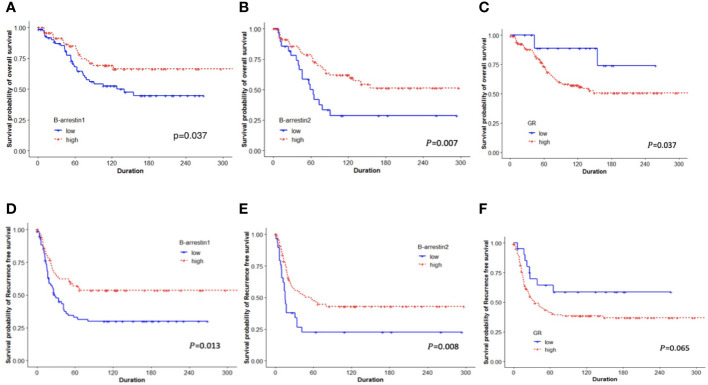
Kaplan–Meier plots for OS and RFS based on categorized β-arrestin 1 and 2 and GR expression. **(A, B)**, Low β-arrestin 1 and 2 expression was associated with shorter OS (log-rank *P* = 0.037, *P* = 0.007, respectively). **(C, F)**, Low GR expression was associated with better OS (log-rank *P* = 0.037), but not with RFS (log-rank *P* = 0.065). **(D, E)**, Patients with low expression of β-arrestins 1 and 2 had significantly shorter RFS than those with high expression (log-rank *P* = 0.013, *P* = 0.008).

The RFS and OS according to β-arrestin 1 and 2 expression levels were determined using Kaplan–Meier curves. The group with high β-arrestin 1 expression had significantly better RFS (*P* = 0.013) and OS (*P* = 0.037) than the group with low β-arrestin 1 expression (**Figures 2A, D**). Similarly, the group with high β-arrestin 2 expression had significantly better RFS (*P* = 0.008) and OS (*P* = 0.007) than the group with low β-arrestin 2 expression (**Figures 2B, E**). Consistent with previous study’s findings, the group with high GR expression demonstrated significantly worse RFS (*P* = 0.037) and OS (*P* = 0.065) than the group with low GR expression (**Figures 2C, F**).

A Cox regression analysis was performed to examine whether β-arrestins were independent factors for prognostication. High β-arrestin 1 expression was a significant prognostic factor for RFS (univariate HR: 0.537, 95% CI: 0.340–0.849, *P* = 0.008; multivariate HR: 0.459, 95% CI: 0.275–0.765, *P* = 0.003). High β-arrestin 2 expression was an independent prognostic factor indicating a better prognosis (univariate HR: 0.507, 95% CI: 0.302–0.8505, *P* = 0.010; multivariate HR: 0.456, 95% CI: 0.263–0.789, *P* = 0.0051).

### 3.6 Prognostic significance of the combination of β-arrestins and the GR

**Figure 3:**
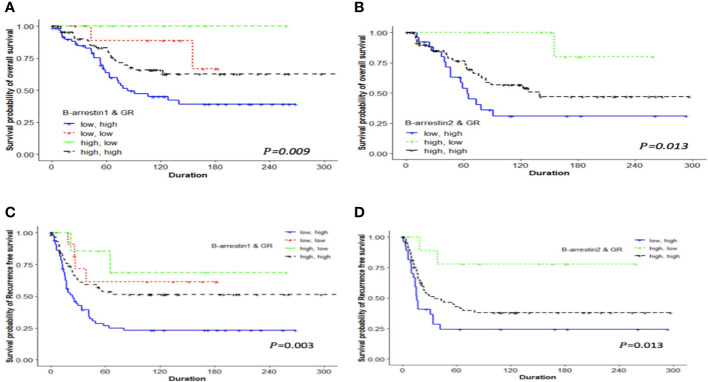
Kaplan–Meier analysis of patients with ovarian cancer based on the combination of β-arrestin 1, β-arrestin 2, and GR expression. OS and RFS differences were observed among the four groups classified according to high or low expression levels. **(A)**, The OS of patients with low β-arrestin 1 expression and high GR expression was shorter (median 63 months) than that of patients with high β-arrestin 1 expression and high GR expression (median 94 months), low β-arrestin 1 expression and low GR expression (median 138.5 months), or high β-arrestin 1 expression and low GR expression (median 85 months) (log-rank *P* = 0.009). **(B)**, Patients with low β-arrestin 2 expression and high GR expression had a significantly shorter OS (median 64 months) than those with high β-arrestin 2 expression and high GR expression (median 140 months) or high β-arrestin 2 expression and low GR expression (median not reached) (log-rank *P* = 0.013). **(C)**, The RFS of patients with low β-arrestin 1 expression and high GR expression was significantly shorter (median 18.5 months) than that of patients with high β-arrestin 1 expression and high GR expression (median 52 months), low β-arrestin 1 expression and low GR expression (median 106 months), or high β-arrestin 1 expression and low GR expression (median 66 months) (log-rank *P* = 0.003). **(D)**, Patients with low β-arrestin 2 expression and high GR expression had a significantly shorter RFS (median 16 months) than those with high β-arrestin 2 expression and high GR expression (median 35 months) or high β-arrestin 2 expression and low GR expression (median 118.5 months) (log-rank *P* = 0.013).

We examined differences in the survival rate based on the expression of β-arrestins 1 and 2 and the GR. After classifying the data into four groups based on expression levels, differences in the survival rate were compared using Kaplan–Meier curves. The survival rate was the lowest when β-arrestin 1 expression was low and GR expression was high, and the highest when β-arrestin 1 expression was high and GR expression was low (log-rank *P* = 0.009 for OS, log-rank *P* = 0.003 for RFS) (**Figures 3A, C**). Similarly, the survival rate was the lowest when β-arrestin 2 expression was low and GR expression was high, and the highest when β-arrestin 2 expression was high and GR expression was low (log-rank *P* = 0.013 for both OS and RFS) (**Figures 3B, D**).

We examined the effect of β-arrestin and GR expression levels on prognosis using the Cox regression model. When using the group with low β-arrestin 1 and high GR expression as the reference arm, the risk was lower in the group with high β-arrestin 1 and GR expression (multivariate HR: 0.459, 95% CI: 0.272–0.775, *P* = 0.003). The risk was also lower, albeit not significantly, in the group with high β-arrestin 1 expression and low GR expression (HR: 0.182, 95% CI: 0.025–1.335, *P* = 0.093). When using the group with low β-arrestin 2 and high GR expression as the reference arm, the risk was lower in the group with high β-arrestin 2 and GR expression (multivariate HR: 0.491, 95% CI: 0.277–0.870, *P* = 0.014). The risk was the lowest in the group with high β-arrestin 2 expression and low GR expression (multivariate HR: 0.159, 95% CI: 0.036–0.700, *P* = 0.015) (**Table 2**).

**Table 2:** Associations between prognostic variables and recurrence-free survival in primary epithelial ovarian cancer.

Risk factor	Univariate regression		Multivariate regression	
	HR	*P*	HR	*P*
β-Arrestin 1 (high^a^)	0.537 (0.340–0.849)	0.007*	0.459 (0.275–0.765)	0.002*
β-Arrestin 2 (high^b^)	0.507 (0.302–0.850)	0.010*	0.456 (0.263–0.789)	0.005*
GR (high^c^)	1.943 (0.940–4.018)	0.073	1.549 (0.708–3.386)	0.273
β-Arrestin 1 low & GR high	Ref.		Ref.	
β-Arrestin 1 high & GR high	0.485 (0.301–0.780)	0.002*	0.459 (0.272–0.775)	0.003*
β-Arrestin 1 low & GR low	0.323 (0.116–0.897)	0.030*	0.525 (0.185–1.492)	0.226
β-Arrestin 1 high & GR low	0.224 (0.054–0.924)	0.038*	0.182 (0.025–1.335)	0.093
β-Arrestin 2 low & GR high	Ref.			
β-Arrestin 2 high & GR high	0.485 (0.301–0.780)	0.002*	0.491 (0.277–0.870)	0.014*
β-Arrestin 2 low & GR low	NA		NA	
β-Arrestin 2 high & GR low	0.224 (0.054–0.924)	0.038*	0.159 (0.036–0.700)	0.015*
Age (≥50 years)	1.481 (0.973–2.254)	0.066	1.905 (1.041–3.487)	0.036*
CA125 (≥35 mmol/l)	2.801 (1.356–5.788)	0.005*	1.105 (0.500–2.442)	0.805
Cell type (serous)	2.583 (1.572–4.245)	0.0002*	1.504 (0.819–2.762)	0.188
Grade (poor)	1.890 (1.247–2.863)	0.002*	1.640 (1.005–2.678)	0.047
Chemosensitivity (resistant)	17.154 (9.371–31.404)	<0.0001*	16.398 (7.442–36.135)	<0.0001*
FIGO Stage (≥III)	6.122 (3.249–11.536)	<0.0001*	3.726 (1.819–7.634)	0.0003*

^a^cut-off of β-arrestin 1 expression = 31.96 established by the Contal & O’Quigley method, ^b^cut-off of β-arrestin 2 expression = 69.32 established by the Contal & O’Quigley method, ^c^cut-off of GR expression = 6.85 according to [(12)].

*P < 0.05.

Ref, reference arm; NA, not applicable.

### 3.7 Predictive power of β-arrestins and the GR

**Figure 4:**
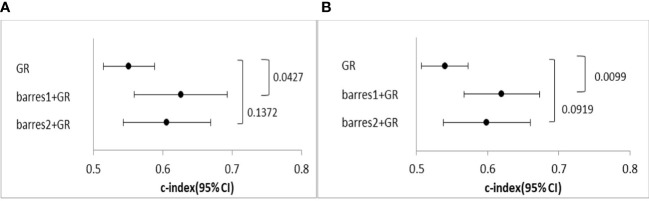
Comparison of the predictive power of the GR and the GR and β-arrestin combination. **(A)**, C-index of univariate Cox regression analysis for OS. **(B)**, C-index of univariate Cox regression analysis for PFS. The GR and β-arrestin 1 expression combination showed a better predictive power than GR expression alone. However, the GR and β-arrestin 2 expression combination showed similar predictive power as GR expression alone.

Harrell’s C-index analysis was performed to determine whether the combination of β-arrestins and the GR improved prognostication. The combination of β-arrestin 1 and the GR demonstrated greater predictive power in terms of both RFS and OS than the GR alone (C-index for β-arrestin 1 and GR = 0.626, 95% CI: 0.559–0.693, *P* = 0.0427; C-index for GR = 0.600, 95% CI: 0.567–0.673, *P* = 0.009). Interestingly, the addition of β-arrestin 2 expression did not significantly affect the prognostic value of GR expression (C-index for β-arrestin 2 and GR = 0.606, 95% CI: 0.543–0.669, *P* = 0.137; C-index for GR = 0.599, 95% CI: 0.538–0.660, *P* = 0.091) (**Figures 4A, B**).

## 4 Discussion

In general, the expression signatures of specific genes or proteins can not only explain the development of certain diseases, but also be utilized as diagnostic biomarkers and treatment targets. The present study demonstrated that the expression of β-arrestin 1 was higher in ovarian cancer specimens than in normal tissues. Furthermore, the combination of β-arrestin 1 and GR expression had a greater predictive prognostic value than GR expression alone, suggesting that β-arrestin 1 may potentially serve as a biomarker in clinical practice and as a therapeutic target. Although further studies are needed to elucidate whether targeting β-arrestin can actually result in improved outcomes, the present study highlights the contribution of β-arrestins in the pathophysiology of ovarian cancer.

Hormones contribute to cancer incidence and mortality; they exert a considerable effect on gynecologic cancers. It is well established that hormones released from the hypothalamic-pituitary-ovarian axis can stimulate or suppress ovarian cancer progression (8, 26, 27). Gonadotropins, estrogens, and androgens may promote ovarian cancer progression, whereas gonadotropin-releasing hormone and progesterone may protect against ovarian cancer (8, 28, 29). Empirical studies have corroborated that hormone receptors are expressed in both normal and ovarian cancer surface epithelium (30) and they may be associated with carcinogens and result in ovarian cancer (31, 32). Although clinical trials using hormonal therapeutic agents in patients with ovarian cancer have been conducted (33, 34), their results have been inconclusive.

Hormone receptors are transcription factors that regulate diverse physiological functions and have decisive roles in hormone-driven cancers. For example, the estrogen receptor (ER) is expressed in 50%–88% of all breast cancers and therefore has served as a primary therapeutic target (35, 36). ER antagonists have demonstrated excellent curative efficacy in patients with ER-positive breast cancer (37); while ovarian cancer is also hormone-driven, the expression and role of hormone receptors in its pathogenesis remain poorly elucidated. We previously assessed the expression levels of ER-α, ER-β, AR, GR, and PR in ovarian cancer tissues and determined their association with the survival rate. The GR was more highly expressed in ovarian cancer tissues than in the non-adjacent normal tissues, and elevated GR levels were associated with a poor prognosis. The GR also affected the expression of the AR and PR (12). High GR expression is associated with an increased risk of disease progression in gynecological and untreated, early-stage, triple-negative breast cancer (38). GR actions can be regulated in a cell type-specific manner, and GR may be a useful therapeutic target in cancer treatment. Upon steroid binding, the GR undergoes activation, dissociates from the chaperone complex, and exerts its various effects (39). The latter requires the translocation of the receptor to the nucleus, where it can bind to glucocorticoid-responsive elements or tether with other transcription factors (40, 41), resulting in a cross-talk between the receptor and an array of elements, such as co-modulators, co-activators, co-repressors, and DNA-remodeling factors (13, 42). β-arrestin 1 has been recently shown to bind to the GR to stabilize the GR protein (16). Accordingly, loss of β-arrestin 1 increases GR protein turnover by promoting its degradation (13).

β-arrestin has been shown to play a key role in various signaling pathways. The binding of β-arrestin 1 to the endothelin-1 (ET-1)/ET A-type receptor (ETAR) signaling complex, and the activation of β-catenin promote the migration, invasion, and progression of ovarian cancer cells (43). Furthermore, β-arrestins activate NF-kB in ovarian cancer cells, thereby enhancing their survival. It has also been reported that β-arrestin 1 is involved in the activation of the YAP/mutant p53 complex in high-grade serous ovarian cancer cells and is associated with increased cancer cell proliferation (44, 45). ETAR antagonists (e.g., ZD4054) can inhibit metastatic progression by interfering with β-arrestin 1 signaling (46). Although the roles of β-arrestin in various signaling systems are being actively studied, there are only a few studies on its survival implication and clinical significance in patients with ovarian cancer.

Therefore, we investigated the relationship between β-arrestin expression and the survival of patients with ovarian cancer. Notably, we found a positive relation between β-arrestin expression levels and the survival of patients with ovarian cancer. Therefore, TCGA data were used to analyze the expression of β-arrestin genes *ARRB1* and *ARRB2* and the survival of patients with ovarian cancer. OS was more favorable when β-arrestins 1 and 2 were highly expressed, whereas lower expression resulted in high RFS (**Supplementary Figure S4**).

These results have also been reported in other cancer types. In lung cancer, high β-arrestin 1 expression was associated with a poor prognosis (20), whereas low β-arrestin 2 expression was associated with a poor prognosis as β-arrestin 2 inhibits lung cancer metastasis (47). In colorectal cancer, high β-arrestin 2 expression inhibits NF-kB activation and is associated with a favorable prognosis (48). β-arrestin 1 has also been shown to play a decisive role in colorectal cancer metastasis by forming a signaling complex with prostaglandin E and c-Src (49). Therefore, it is important to elucidate the tissue-specific roles of β-arrestins and their interactions with key signaling cascades in each tumor type.

In conclusion, we evaluated the expression of β-arrestin in ovarian cancer tissues and observed that its increased expression was associated with a good prognosis and had clinical significance in epithelial ovarian cancer. However, it should be noted that this was a retrospective study with several inherent biases and limitations, such as a low number of patients and a broad timeline of inclusion of cases. Additionally, no cellular experiments were conducted to validate our results. Despite these limitations, our study suggests that β-arrestin, in combination with the GR, may have enhanced predictive power for patients with epithelial ovarian cancer, implicating a possible role in prognostication.

A marker that can predict prognosis or treatment response can be an essential clue to providing more differentiated and effective treatment for each individual. There is a need for more powerful markers for the treatment of ovarian cancer. In oncology, efforts have been made to identify non-invasive and more efficient markers. Recently, 2-^18^FDG PET/CT has been known to have recurrence detection and prognostic values, and expectations for their role as an imaging biomarker are growing (50, 51). Attempts to combine new biomarkers with clinical features or imaging technique provide an opportunity to predict the patient’s prognosis and offer improved individualized treatment. This potential was observed in the case of the epithelial ovarian cancer biomarkers, β-arrestin and GR, in this study.

## Data availability statement

The datasets presented in this study can be found in online repositories. The names of the repository/repositories and accession number(s) can be found in the article/**Supplementary Material**.

## Author contributions

Conceptualization: J-WR, GH, and J-HK. Data curation: J-WR, H-SK, JK, H-NL, and J-YC. Methodology: J-WR, J-YC, and J-HK. Funding acquisition: HC, J-HK. Resource: GH, H-SK. Visualization: H-Y.S, J-YC. Writing-original draft: J-WR. Writing-review and editing: J-HK, J-YC, and J-WR. Supervision: HBC, J-HK. All authors have read and agreed to the published version of the manuscript. All authors contributed to the article and approved the submitted version.
