# Supplementary material for: Prognostic value of β-Arrestins in combination with glucocorticoid receptor in epithelial ovarian cancer
Source: Front Oncol. 2023 Mar 10;13:1104521. doi: 10.3389/fonc.2023.1104521 (PMC10036403; doi:10.3389/fonc.2023.1104521)

**Table S1:** Clinicopathologic characteristics of patients

|  |  | N | % |
| --- | --- | --- | --- |
| Age | $\leq$50 | 74 | 43.5 |
|  | >50 | 95 | 55.9 |
| Ca125 | $\leq$35 | 28 | 16.6 |
|  | >35 | 141 | 83.4 |
| Cell differentiation | well | 15 | 9 |
|  | moderate | 70 | 41.9 |
|  | poorly | 82 | 49.1 |
| Cell type | serous | 111 | 65.7 |
|  | endometrioid | 23 | 13.6 |
|  | Mucinous | 20 | 11.8 |
|  | Clear cell | 11 | 6.5 |
|  | Transitional | 4 | 2.4 |
| Chemosensitivity | sensitive | 131 | 77.1 |
|  | resistant | 23 | 13.5 |
| Stage | I | 41 | 24.1 |
|  | II | 15 | 8.9 |
|  | III | 98 | 58.0 |
|  | IV | 14 | 8.9 |
| Status | alive | 108 | 63.9 |
|  | expire | 61 | 36.9 |
| Recurrence |  | 93 | 54.7 |

**Figure S1:** Expression level according to stage

A . β-arrestin 1 B. β-arrestin 2


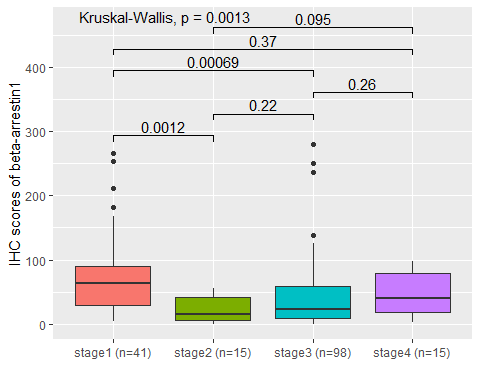

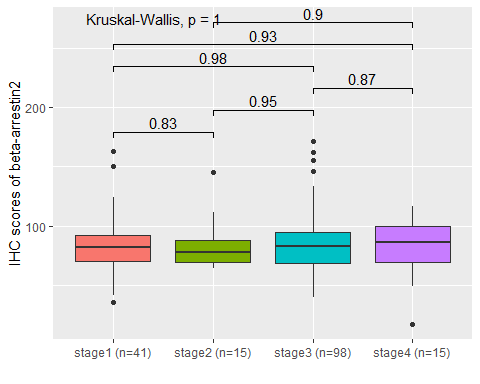


C. Glucocorticoid receptor


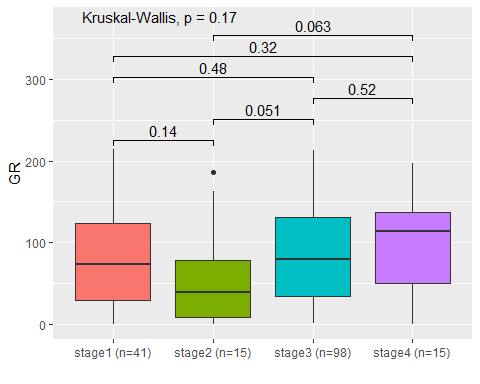


**Figure S2:** GEO datasets

A. GSE14407


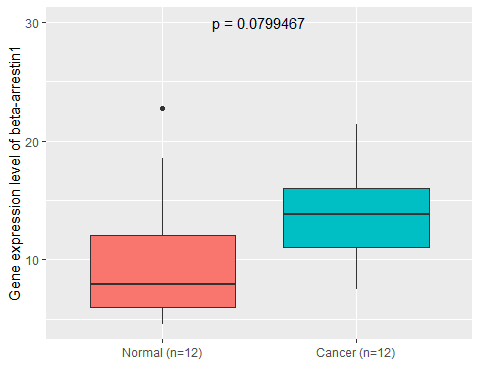

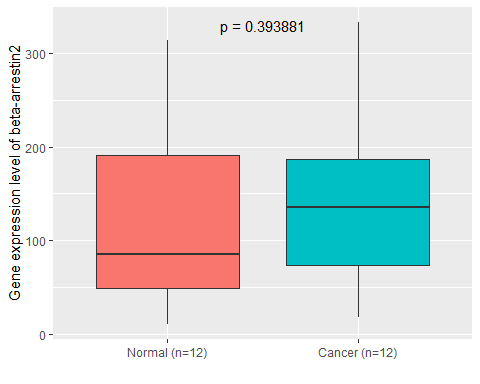


B.GSE26712


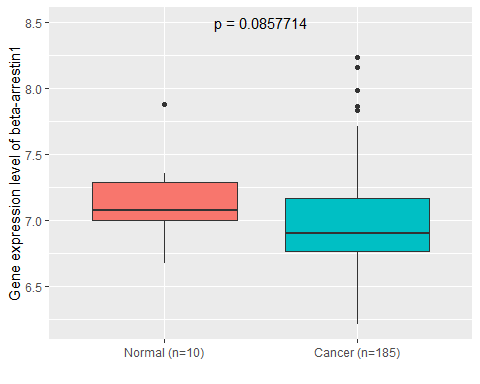

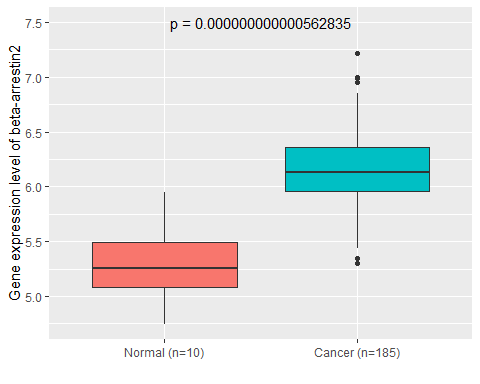


C.GSE 16570


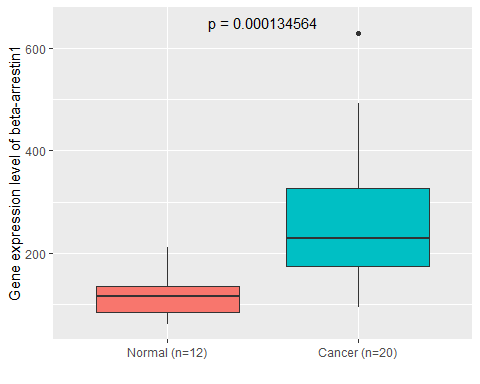

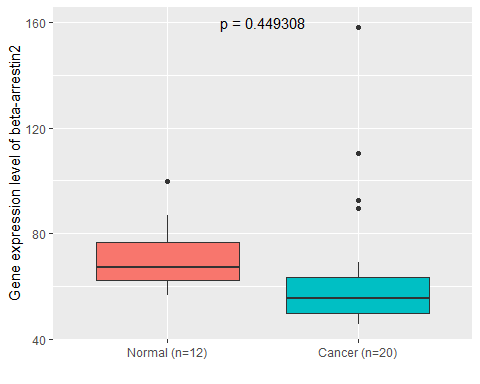


**Figure S3:** Correlation of GR and β-arrestin1,2

1. B.


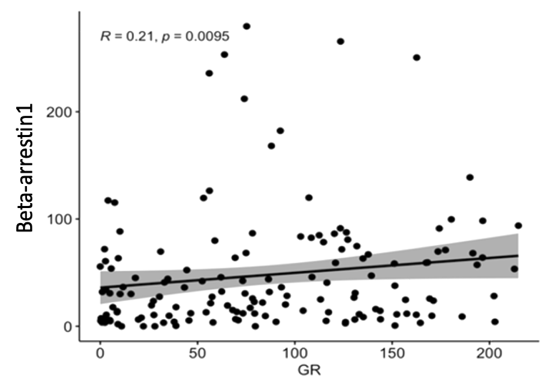

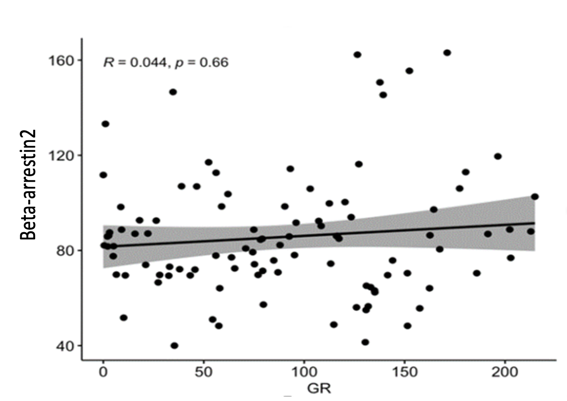


Correlation of β-arrestins 1 and 2 and GR: Spearman correlation. Moderate correlation with β-arrestin. 1 and GR (A); β-arrestin 2 showed a positive correlation with GR expression and had no statistical significance (B).

Figure S4: GEPIA data


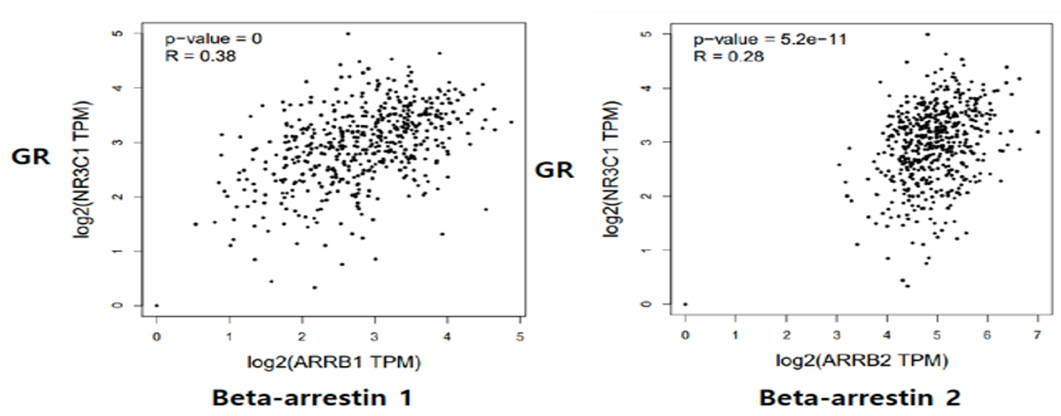


**Figure S5:** TCGA data (2011, nature)


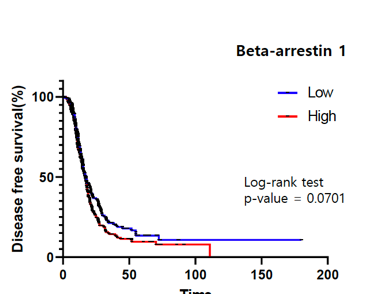

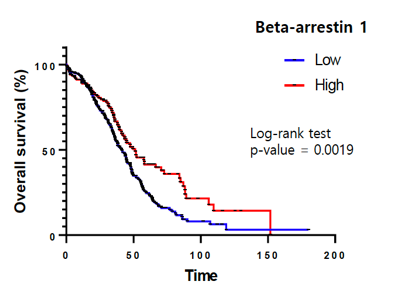


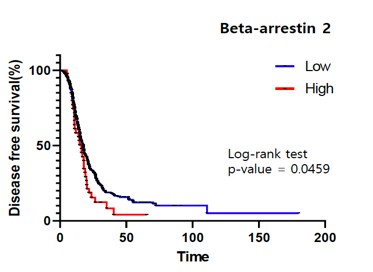

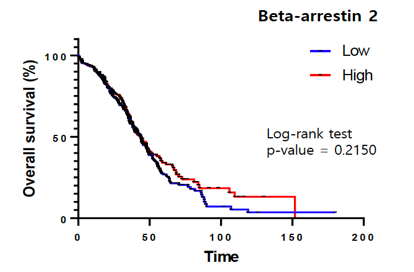

Supplement: Supplementary file 1 [file DataSheet_1.docx]
